# Methylation status of oestrogen receptor-α gene promoter sequences in human ovarian epithelial cell lines

**DOI:** 10.1038/sj.bjc.6600028

**Published:** 2002-01-21

**Authors:** A M O'Doherty, S W Church, S E H Russell, J Nelson, I Hickey

**Affiliations:** School of Biology and Biochemistry, The Queen's University of Belfast, Lisburn Road, Belfast, Northern Ireland, UK; Department of Oncology, The Queen's University of Belfast, Lisburn Road, Belfast, Northern Ireland, UK

**Keywords:** oestrogen receptor, DNA methylation, ovarian cancer

## Abstract

We have determined the methylation status of the CpG island of the oestrogen receptor α gene in seven human ovarian cell lines. Cell lines expressing oestrogen receptor α showed no evidence of hypermethylation. In three of four cell lines that produced no detectable oestrogen receptor α protein, hypermethylation was observed at the *NotI* site of the CpG island. These results indicate that aberrant hypermethylation may be responsible for a significant proportion of epithelial ovarian tumours in which oestrogen receptor α expression is lost.

*British Journal of Cancer* (2002) **86**, 282–284. DOI: 10.1038/sj/bjc/6600028 www.bjcancer.com

© 2002 The Cancer Research Campaign

## 

The expression of oestrogen receptor α (ER-α) detected in the epithelial cells of normal human ovaries is frequently lost in ovarian cancers (Lau *et al*, 1999). ER-α expression is significant in epithelial ovarian cancer as, in conjunction with progesterone receptor, it is reported as a good prognostic factor (Leake and Owens, 1990). Silencing of a number of different genes in tumours often arises through hypermethylation of promoter regions. In ovarian cancer methylation has been reported for a number of loci including BRCA1 (Baldwin *et al*, 2000; Strathdee *et al*, 2001). The CpG island 5′ of the ER-α gene has been found to be hypermethylated in a high proportion of ER-negative breast tumours (Lapidus *et al*, 1996) and in breast cell lines (Ottaviano *et al*, 1994). This has also been reported in colorectal tumours (Issa *et al*, 1994) and several other cancers (Issa *et al*, 1996; Li *et al*, 2000).

We have examined ER-α expression and methylation of the ER-α CpG island in a series of human epithelial ovarian cell lines using the technique of methylation specific polymerase chain reaction (MS–PCR) (Herman *et al*, 1996). This allows rapid and unambiguous determination of the methylation status of the regions of DNA complementary to the PCR primers used.

## MATERIALS AND METHODS

### Cell lines

PEO 1, PEO 4, PEA 1 and PEO 14 were gifts of Dr S Langdon, ICRF Edinburgh, UK. OAW42 was obtained from Dr A Wilson, Derby City Hospital, UK. OTN14 was supplied by Dr M Cairns, Department of Oncology, Queen's University Belfast, UK. All cell lines were maintained in RPMI 1640 (Life Science Technologies) supplemented with 2.4 μg ml^−1^ insulin and 10% foetal calf serum (PAA), in an atmosphere of 5% CO_2_.

### Western blotting

A sub-confluent culture from a 75 cm^2^ flask was used for protein extraction. Cells were scraped into 1.0 ml PBS, pelleted and frozen at −70°C. The pellet was thawed into 200 μl high salt buffer (0.4 M KCl, 20 mM HEPES, 20% (v v^−1^) glycerol, 1 mM dithiothreitol, 40 μg ml^−1^ PMSF and 20 μl ml^−1^ protease inhibitor cocktail (Sigma). It was then syringed through a 26G needle 10–15 times and microfuged at 13 000 r.p.m. for 20 min. The supernatant was stored at −70°C. Protein was quantified by the BCA method (Pierce). Proteins were electrophoresed in vertical 12% polyacrylamide gels, at a constant current of 30 mA for 2–4 h, and were subsequently transferred to Protran nitrocellulose using a Mulitphor II semi-dry blot apparatus (Pharmacia), at 1 mA per cm^2^ for up to 2 h. Filters were blocked using 5% Marvel milk powder, and ER-α detected using 1 : 200 dilutions of murine antibody to human oestrogen receptor (Dako). After washing, 1 : 500 dilutions of goat-anti mouse secondary antibodies were added for 1 h at room temperature. The bound antibodies were then visualized by enhanced chemiluminescence using the Amersham ECL kit as described by the manufacturer's instructions. Restripping of blots was achieved by immersion in a solution containing 100 mM β-mercaptoethanol, 2% (w v^−1^) SDS, 62.5 μM, Tris-HCl pH 6.7, for 30 min at 60°C with agitation. Tubulin detection on blots was carried out as for ER-α except that antibody against tubulin (Dako) was used.

### MS–PCR

After treatment with Na bisulphite to induce deamination of methylated cytosines MS–PCR was carried out by a modification of the method described by Lapidus *et al* (1996). The primers used were designed to detect methylation at the *NotI* site of the ER-α promoter. The sequence of the primers for unmethylated DNA were 5′ TGTTGTTTATGAGTTTAATGTTGTGGTT 3′ and 5′ AAAAAAACCCCCCAAACCATT 3′. These gave a product of 124 bp. For methylated DNA the primers were 5′ ACGAGTTTAACGTCGCGGTC 3′ and 5′ ACCCCCCAAACCGTTAAAAC 3′ which gave a product of 110 bp (J Herman, private communication).

The MS–PCR conditions used for both methylated and unmethylated primers were identical. After an initial denaturation at 95°C for 5 min *Taq* DNA polymerase was added and the reaction continued for 35 cycles each consisting of denaturation at 95°C for 30 s, annealing at 57°C for 30 s and extension at 70°C for 30 s. A final extension at 72°C for 5 min was followed by storage at 4°C overnight. Products were visualized after electrophoresis in a 2% agarose gel for 1.5 h at 80 V.

## RESULTS

**Figure 1:**
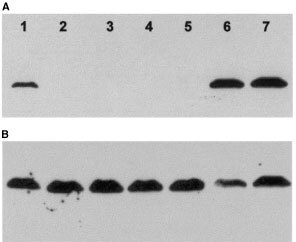
(**A**) Western blot of protein extracts from ovarian cell lines probed with antibody against ER-α Lanes: 1, PEO1; 2, PEO14; 3, A2780; 4, OTN14; 5, OAW42; 6, PEO4; 7, MCF-7. (**B**) The same blot stripped and reprobed with antibody against tubulin.

We analyzed the ER-α status of six epithelial ovarian cell lines by Western blotting. The well-characterized breast cancer cell line MCF-7 was used as a positive control. The data presented in Figure 1A

clearly indicate that of the ovarian cell lines only PEO1 and PEO4 display a positive signal for the ER-α protein. These two cell lines were derived from the same patient at different stages of her treatment (Langdon *et al*, 1988), and thus it is not surprising that they share the same ER-α phenotype. In each case only a single band of 67 kDa was observed and of identical mobility to the band present in MCF-7. The levels of expression were similar in PEO 4 and MCF-7, but reduced in PEO1. The ovarian cell lines PEO14, A2780, OTN14 and OAW 42 were clearly negative for expression of ER-α protein. The blots were subsequently stripped and reprobed with tubulin antibody. Figure 1B shows that there was no evidence of protein degradation in ER-α negative cell lines and approximately equal loading of protein in each lane.

**Figure 2:**
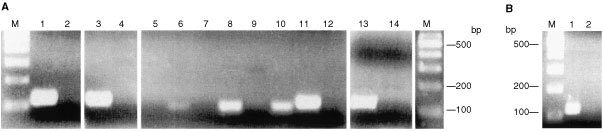
(**A**) MS–PCR of Na bisulphite-treated DNA from cell lines. Lanes 1, 2: PEO 1; lanes 2, 3: PEO 14; lanes 5, 6: A2780; lanes 7, 8: ONT 14; lanes 9, 10: OAW 42; lanes 11, 12: PEO 4; lanes 13, 14: MCF-7. Unmethylated reactions: lanes 1, 3, 5, 7, 9, 11 and 13. Methylated reactions: lanes 2, 4, 6, 8, 10, 12 and 14. M, 100 bp marker. (**B**) MS–PCR of Na bisulphite treated DNA from cell line PEA 1. Lane 1, unmethylated reaction; lane 2, methylated reaction; M, 100 bp ladder.

We then assessed whether the loss of ER-α expression in the cell lines was associated with DNA methylation in the CpG island of the ER gene using MS–PCR with primers designed to identify the *NotI* site of the ER-α promoter. The results of these amplifications are shown in Figure 2

. Products of MS–PCR for each cell line were electrophoresed in adjacent pairs of lanes. The left-hand lane represents the product of the primers designed to amplify unmethylated DNA and the right-hand lane the product from primers for methylated DNA. Each cell line produced product in only one lane. The two ER-α positive ovarian cell lines and MCF-7 all produced bands only for the left-hand lanes and are clearly unmethylated as would be predicted. Of the four ER-α negative ovarian cell lines OAW42, OTN14 and A2780 each gave a product only with the primers designed to detect methylated DNA. PEO14 however only gave product with primers for unmethylated DNA.

In addition we examined the methylation status of the ovarian cell line PEA1, that has been reported as expressing ER-α at very low levels (Langdon *et al*, 1990). As shown in Figure 2B it is unmethylated at this locus.

## DISCUSSION

It would appear that DNA methylation of promoter sequences is associated with non-expression in three of the four ER-α negative epithelial ovarian cell lines. In the case of PEO14 the loss of ER-α expression may be due to other causes such as mutation, or suppression through reorganization of chromatin that is independent of methylation. We have found it impossible to induce re-expression of ER-α in this cell line by treatment with the inhibitors of DNA methyltransferase, 5-azacytidine or 5-aza-deoxcytidine. This supports the suggestion that DNA methylation is not responsible for the ER-negative phenotype in this cell line.

Since PEA1 only expresses ER-α at very low levels, it would appear that lack of methylation of the ER-α GpG island is necessary for expression of the gene but other factors quantitatively regulate the level of expression of the gene. Of the three cell lines that show methylation of the ER-α promoter OAW42 derives from serous (Wilson *et al*, 1984), and OTN14 ( van Niekerk *et al*, 1988) from mucinous tumours. Indicating that promoter methylation is not specific to individual ovarian cancer pathologies. The pathology of the tumour from which A2780 was derived is not known (Behrens *et al*, 1987). In culture the three cell lines show no other linking phenotypes except that all lack mutations in p53. It has been suggested that tumours displaying hypermethylation of GpG islands have reduced frequencies of mutation at p53 (Toyota *et al*, 2000).

A question can be raised as to the contribution of the loss of ER-α expression to the malignant phenotype of the ovarian tumour cell lines. This stems from the fact that loss of ER-α expression through hypermethylation of promoter sequences is known to increase with age (Toyota and Issa, 1999). As ovarian tumours arise predominantly in women over 50 years of age it is possible that methylation of the ER-α gene preceded the development of tumorigenesis. It should be noted however that none of the three negative cell lines were heterozygous for methylation of the ER-α gene. This may be the result of loss of one copy of the gene or because the sequence of the ER-α promoter acts as a favoured site for aberrant DNA methylation in tumour cells. The functional significance of the inactivation of ER-α will only be determined by analyzing the growth properties of cells in which the gene has been reactivated by treatment with inhibitors of DNA methyltransferase or histone deacetylase.
